# Perspective Review of Polymers as Additives in Water-Based Fracturing Fluids

**DOI:** 10.1021/acsomega.1c06739

**Published:** 2022-02-23

**Authors:** Sameer Al-Hajri, Berihun M. Negash, Md Motiur Rahman, Mohammed Haroun, Tareq M. Al-Shami

**Affiliations:** † Petroleum Engineering Department, Khalifa University, P.O. Box, 127788 Abu Dhabi, United Arab Emirates; ‡ Department of Petroleum Engineering, Universiti Teknologi PETRONAS, Persiaran UTP, 32610 Seri Iskandar, Perak, Malaysia

## Abstract

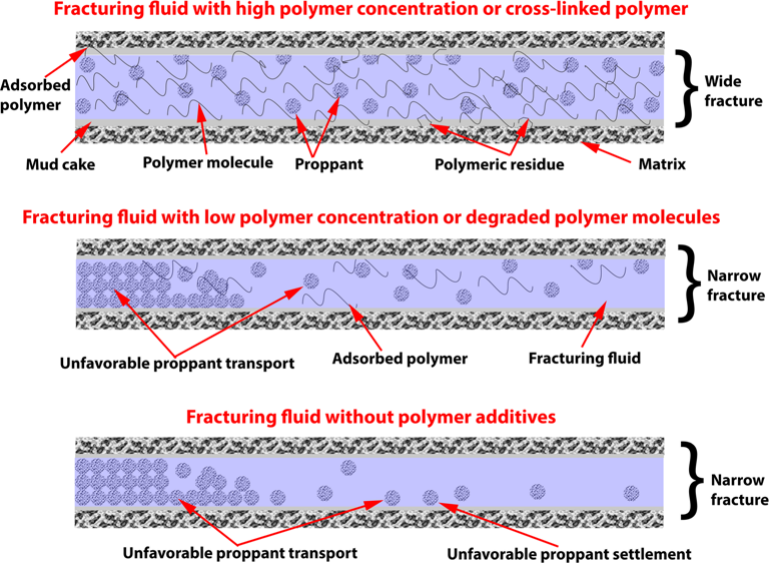

After successful implementation for more than 6 decades by the oil and gas industry, hydraulic fracturing remains the pioneer well stimulation method to date. Polymers are one of the additives in fracturing fluids that play a significant role. Polymers are used as friction reducers and viscosifiers to provide a transport medium for proppants in fracturing fluids. There are many polymer-based fracturing fluid systems, but choosing the most appropriate type and system depends on the type of application and a wide range of parameters. Currently, there is no complete review study that gives a reference and hence a perspective for researchers on the use of polymers in hydraulic fracturing. This paper summarizes the published literature on polymers used in fracturing fluids and discusses the current research issues, efforts, and trends in the field, aiming to provide an overview of the polymer applications in slick-water and cross-linked gel systems. The mechanism and limitation of polymer use such as polymer degradation, fracture conductivity reduction, and polymer adsorption are also reviewed in this paper. The reviewed literature suggested that polymers are important additives in fracturing fluids not only to provide adequate transportation of proppants but also to determine the width of the fracture whereby higher viscosities yield wider fractures. The development of synthetic polymers and associative polymers in fracturing fluids showed a remarkable potential to improve the stability of fracturing fluids in unconventional reservoirs under reservoir conditions, which makes it an interesting topic for future studies.

## 1 Introduction

**Figure 1:**
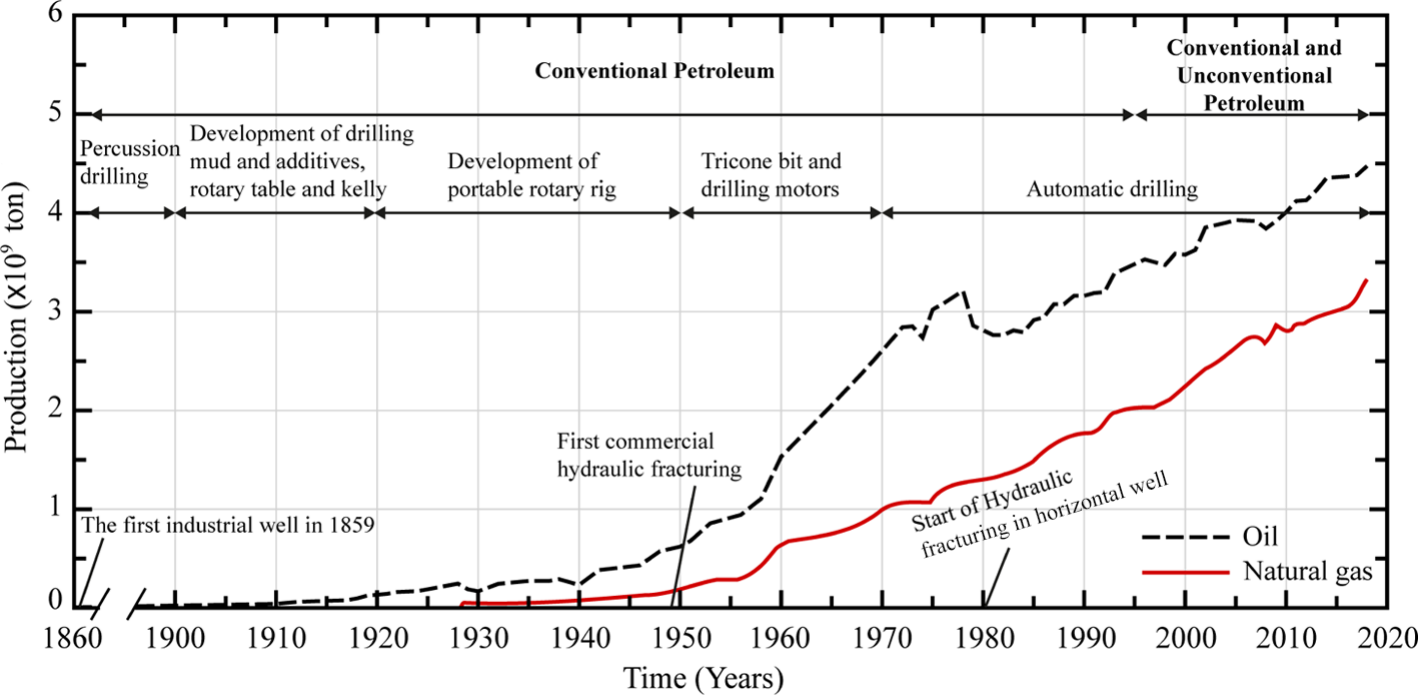
Oil and gas production development over the years.

The first industrial oil well was drilled in Pennsylvania, USA, in 1859.^1^ Since then, exploration and development slowly and gradually increased until a significant increase in oil and gas production was observed in 1920. The increase amounts to approximately 0.11 × 10^9^ tons of oil equivalent, as shown in Figure 1. Further understanding of petroleum geology and accumulation conditions of hydrocarbons from the 1920s to the 1950s led to the development of the tricone bits and the evolution of rotary drilling, especially the use of drilling motors. By 1955, the equivalent oil and gas production jumped to 1.05 × 10^9^ tons. The theories of oil generation, petroleum geology, and horizontal drilling continued to develop significantly from the 1960s to the 1990s, resulting in a rapid increase in oil and gas production, reaching more than 5.2 × 109 tons of oil equivalent in 1995. Hydrocarbon production technologies were extensively applied after 1995 by using high-resolution seismic imaging, fine reservoir characterization, and numerical simulations of petroliferous systems. As a result, production has increased significantly over the past decade.

The advancement of multistage horizontal drilling and the decline in conventional hydrocarbon reserves led to progress in unconventional oil and gas exploration and development. This resulted in an increased hydrocarbon production of approximately 8 × 10^9^ tons by 2020. Currently, the oil and gas industry has stepped deeply into unconventional resources, which are proven to hold a significant amount of untapped hydrocarbon resources. Now, shales have become the main source of unconventional resources, which was not predicted 35 years ago.^2^

### 1.1 Hydrocarbon Production from Shale

Shales are unconventional geological rocks composed mainly of clay-size mineral grains (illite, kaolinite, and smectite), typically formed by the deposition of fine sediments at the bottom of seas or lakes in fairly quiet environments. Shale may contain other minerals such as quartz, chert, and feldspar.^3^ Shale formations are the most prolific sedimentary rocks in the earth’s crust. Shale formations play a major role in hydrocarbon recovery because they can serve as hydrocarbon source rocks, seals, and reservoirs in shale oil/gas plays, all at the same time.

Shale has ultralow porosity and permeability, and the results of experiments on core analysis show that shale rock permeability is mainly less than 150 nD with pore throat diameters of 4–200 nm.^4^ It is also reported that gas is stored in shale gas reservoirs typically in three forms:^5^ (1) free gas mainly in the nanopores, (2) dissolved gas in the organic kerogen,^6^ and (3) adsorbed on the surface of the rock. The extremely low permeability of the shale with pore sizes in the nanoscale makes Darcy’s law inapplicable for interpreting the gas flow in shale reservoirs.^6^ The flow of hydrocarbons in shale formations is complex and not fully understood involving various mechanisms including slip flow, Knudsen diffusion, viscous flow, and gas adsorption/desorption.^5^ To improve the understanding on flow behavior in complex formation such as shales, profound studies were conducted, resulting in the development of quasi-static, numerical, and analytical reservoir models for unconventional reservoirs.^7^ Further modeling work including compositional modeling work on fluid flow through shale hydrocarbon reservoirs was also conducted.^8^

### 1.2 Fracturing Shale Reservoirs

**Figure 2:**
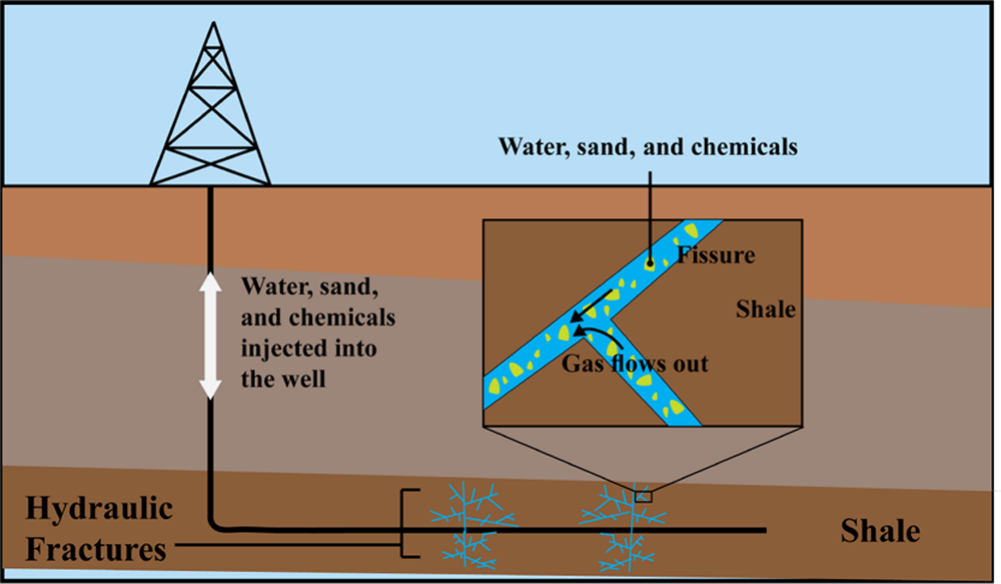
Hydraulic fracturing in shale formations.

Over 60 years ago, Halliburton Oil Well Cementing Company used hydraulic fracturing for the first time to stimulate oil and natural gas wells.^9^ The new method has increased production rates, and the practice quickly spread throughout the world. Currently, hydraulic fracturing is applied to thousands of wells every year. Hydraulic fracturing is a process in which the fluid is pumped into the reservoir through a perforated interval at elevated pressures to break the reservoir rock, creating fractures as illustrated in Figure 2. In shale reservoirs, hydraulic fracturing operation is usually combined with horizontal drilling, which makes natural gas production from unconventional shale gas economically possible. A horizontal section of the well is completed with multiple transverse fractures to increase the stimulated reservoir volumes in shale hydrocarbon reservoirs.

Despite the fact that the shale pores are in the nanoscale, they represent a significant fraction of the shale matrix. As a result, shale rocks ideally hold a considerable volume of oil/gas/water up to 1000 Tcf of the potential natural gas reserve in place.^10^ However, due to the extremely low permeability, these fluids lack adequate means of transport within the rock and to the wellbore. Therefore, induced fractures interacting with the natural fractures create the fracture network which helps the fluids to flow from the matrix to natural fractures and induce fractures simultaneously. Researchers have reported attempts of fracture network creation for the Barnett Shale, showing initial relationships between the shape, network, treatment size, and the production response.^11^

During hydraulic fracturing, some fluid leaks off into the formation with continuous injection at high pressures, but the bulk volume of the fluid inside the fracture maintains enough pressure, allowing the propagation of the fracture, which continues until the net pressure at the fracture tip becomes zero. Initially, a clean fluid (proppant-free) is pumped to create the desired fracture dimensions, and then a mixture of the fluid and proppant is pumped to the open fractures in the formation. Finally, the fluid injection is stopped and the injected fluid is remained in the fracture. The remaining fluid in the fracture starts to leak off into the formation, which depletes the pressure inside the fracture. This results in the closure of the fractures on the settled proppant, which creates a conductive path for the hydrocarbon to flow. Then, the remaining fracturing fluid flows back to the well up to the surface. The width and extension of fractures, proper proppant transportation, and pressure drag reduction are the main functions of a fracturing fluid of required rheological properties.

### 1.3 Fracturing Fluids

The fracturing fluid is significant in creating the desired fracture geometry and controlling the carrying efficiency of a proppant; thus, proper selection of the fracturing fluid is vital in hydraulic fracture treatment.

A wide variety of fracturing fluids have been reported in the literature in various methods to satisfy the fracture treatment parameters, which are controllable and some are dependable. Various chemical compositions and types of fracturing fluids have been reported in the literature such as foam fluids, carbon dioxide, nitrogen gas, gelled oils, aqueous solutions of polymers with or without cross-linkers, viscoelastic surfactant (VES) solutions, slick water, and emulsions. Table 1 shows a summary of different fluids used for hydraulic fracturing.^12^

**Table 1:** Different Base Fluids Used for Hydraulic Fracturing

base fluid	fluid type	main composition
water	slick water	water, sand, and a small fraction of chemical additives.
	cross-linked	cross-linker and a polymer such as Guar.
	surfactant gel	electrolyte and surfactant.
	foam	foamed water with a gas such as N_2_ and CO_2_.
foam	acid-based	foamed acid with N_2_.
	alcohol-based	foamed methanol with N_2_.
oil	cross-linked fluid	phosphate ester gels.
	water emulsion	water, oil, and an emulsifier.
	linear	
acid	cross-linked	
	oil emulsion	
	methanol	water with methanol mix or 100% methanol.
emulsion	oil	water and oil emulsion.
	CO_2_	CO_2_, water, and methanol.
	liquefied CO_2_	CO_2_
other fluids	liquefied nitrogen	N_2_
	liquefied helium	He
	liquefied natural gas	LPG (butane and/or propane)

Water-based slick water and gel fracturing methods have proven to be pioneering methods among the various fracturing methods.^13^ Both methods use polymers for either a drag/frictional pressure reduction or improving fluid viscosity to increase the fluid’s ability to suspend and transport the proppants.

## 2 Polymers in Fracturing Fluids

The next three sections in this research will be discussing the mechanisms, applications, and challenges of polymers in fracturing fluids as drag reducers and cross-linked polymer gels. The efforts to mitigate the use of polymers in fracturing fluids will also be discussed.

### 2.1 Mechanisms of Polymers in Fracturing Fluids

Polymers are mainly used in fracturing fluids to reduce the friction in the turbulent flow or to improve the proppant carrying capacity of the fracturing fluid. The mechanism of improving fracturing fluid carrying capacity and reduction of friction is by increasing the viscosity of the fracturing fluid, which becomes non-Newtonian.

#### 2.1.1 Mechanism of Drag Reduction

A very small polymer concentration dissolved in a liquid results in the reduction of fluid friction with the walls of the pipe. However, this is only true for turbulent flows observed in applications such as well stimulation. This is because of the unorganized fluid motion at the edges causing the initiation of a lateral mixing by fluid eddies, resulting in significant turbulent friction and hence consumption of energy at the pipe wall.^14^ The exact mechanism of this drag reduction is complex; however, there are several suggested mechanisms reported in the literature. Toms (1977) explained that drag reduction is a result of the shear-thinning layer near the wall.^15^ Similarly, Han and Choi (2017) reported that shear thickening has a drag reduction ability.^14^

**Figure 3:**
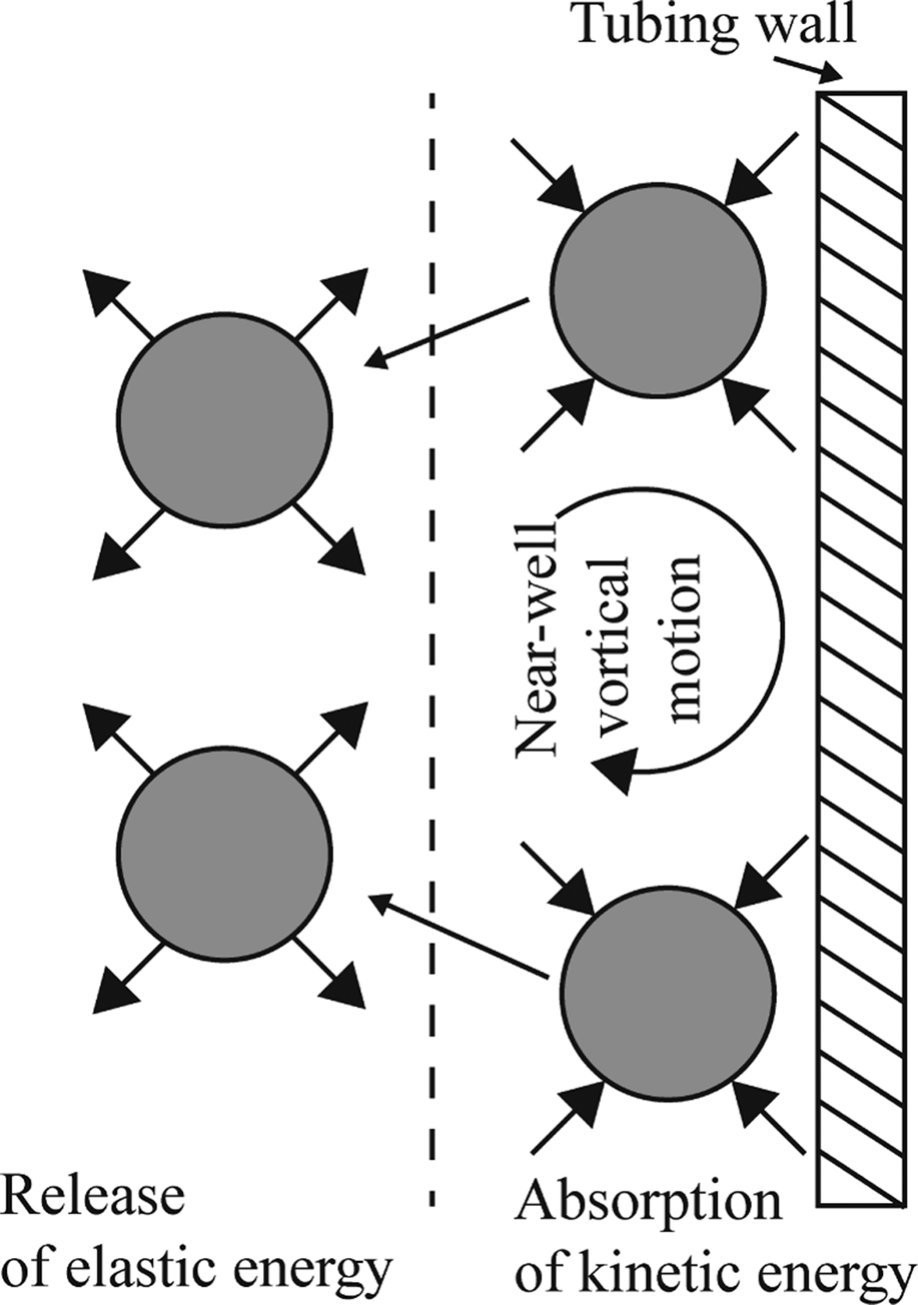
Schematic of the drag reduction mechanism.

The polymer solution drag reduction viscoelastic behavior was also reported as a mechanism for drag reduction. Other mechanisms based on the molecular extension proposed that polymer molecules outside the viscous sublayer can be expanded/stretched, which increases the thickness of the viscous sublayer, decreasing the velocity in the vicinity of the wall.^16^ Another interesting mechanism was proposed by Min et al. (2003), who suggested that fluids with high elastic energy near the tubing wall are transported and pushed away by the vortex near the wall, as shown in Figure 3. If the relaxation time is small enough, the particles (polymer) release the elastic energy near the tubing wall prior to reaching the buffer layer. On the other hand, elastic energy formed by the kinetic energy will reach the buffer and release there, resulting in turbulence weakening near the wall if the relaxation time is sufficiently long.^17^

#### 2.1.2 Mechanism of Increasing the Carrying Capacity

Polymers are chains of monomers with high molecular weight. The addition of a polymer into a liquid will significantly increase the viscosity of the solution. The mechanism of the viscosity increase can be explained by intensive internal friction between the polymer macromolecules which are randomly coiled and the molecules of the surrounding solvent. The polymer type and solvent nature will determine how much the viscosity of a polymer solution can be increased.

Linear polymers can also interconnect at several points on the polymer chains and form a single macromolecule with a network structure. This mechanism is called cross-linking of a polymer. Different techniques are used to cross-link polymers depending on the nature of the polymer. Cross-linking can occur by monomer polymerization (by condensation) or by covalent bonding among polymeric chains by sulfur vulcanization, irradiation, or chemical reactions through the addition of different chemicals (metals) with heating or pressure.^18^ It is noteworthy to mention that regardless of the mechanism at which the polymer is cross-linked, the polymer chemical structure will be altered by the cross-linking process.

**Figure 4:**
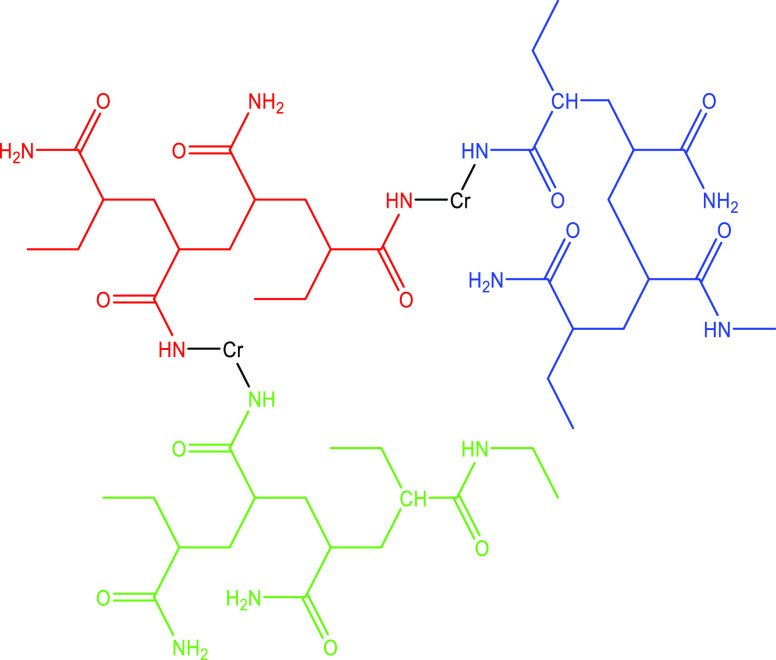
Cross-linked polyacrylamide with chromium metal ions forming a complex network.

The typical polymer concentration used in fracturing fluids to cross-link and create gels is 20–40 pptg.^13^Figure 4 illustrates a cross-linking method of a polyacrylamide polymer containing carboxyl groups, by which polymer chains are connected, forming a cross-linked gelled polymer.^19^

### 2.2 Application of Polymers in Fracturing Fluids

The polymer is a very important additive in hydraulic fracturing. Polymers are mainly used in the water-based fracturing fluid to increase its viscosity. Polymers are used in two forms in hydraulic fracturing: linear gels (drag reducers) and cross-linked gels (transport medium for the proppant).

#### 2.2.1 Polymers as a Drag-Reducing Agent

Drag reducers have been implemented in the field since the mid-1950s due to their ability to exhibit low friction pressure. While flowing in a pipeline, a drag reducer disrupts the near-wall turbulence by interacting directly with the vortex, thereby reducing the friction in the pipeline. Laboratory flow loop tests addressed this mechanism and reported a drag reduction of 10–85% in the lab and 30–90% in the field^20^ compared with that of freshwater.

Earlier studies by Al-Sarkhi et al. (2001a) reported polymer drag reduction in annular liquid flow changing from annular to stratified flow with the addition of low concentration of polymers with high molecular weight.^21^ Al-Sarkhi et al. (2001b) also reported polymer drag reduction at different tubing diameters.^22^ Their results showed up to 65% drag reduction, which resulted in the change in the flow pattern and a significant reduction of friction with the tubing’s walls. Another study by Kang et al. (1998) demonstrated a drag reduction of 35%, allowing oil and water to flow in the pipe with very low concentrations (5–75 ppm) of the polymer.^23^ Similarly, Milligan et al. (2011)^76^ reported 30% drag reduction using a very low concentration of 50 ppm poly(2-ethylhexyl-methacrylate) (PEHMA).^24^ A more recent study by Kotenko et al. (2019) reported a drag reduction of 60–80% at a higher concentration with high fluid velocities ranging from 0.5 to 3.5 m/s.^25^

Drag reducers are added in small quantities to the fracturing fluid and are mostly shear-sensitive, large polymers exhibiting non-Newtonian flow behavior. This requires a higher molecular weight with longer polymer molecules. Table 2 summarizes a typical composition of a water-based fracturing fluid showing a very small but significant amount of polyacrylamide (drag reducer).^20^

**Table 2:** Typical Fracturing Fluid Composition Used for Hydraulic Fracturing

additive	quantity w/w %	component	purpose
water	90.6		the main component in the fracturing fluid. It provides a medium for transporting fracturing additives and hydrostatic pressure.
salt	0.05	potassium chloride	mixed with fresh water or brine to increase salinity to the desired value.
sand	8.95	silica, quartz	mixed with the fracturing fluid to keep the fractures opened to provide a conductive path for the oil/gas to the production well.
iron control	0.004	citric acid	works as a sequestering agent to prevent metal oxide precipitation.
drag Reducer	0.08	polyacrylamide (anionic, cationic, or nonionic)	reduces fracturing fluid friction to withstand desirable injection rates and pressures.
surfactant	0.08	ethoxylated alcohols, isopropanol	increases fracturing fluid viscosity.
breaker	0.009	peroxide, enzyme complexes	mostly, oxidizers/enzymes typically break down the viscosifiers into smaller particles with smaller molecular weights to place the proppant at the fractures and provide cleanup for the fractures to improve the flow to the production well.
biocide	0.001	glutaraldehyde, 2,2-dibromo-3-nitrilopropionamide, tetrakis(hydroxymethyl)phosphonium sulfate, Dazomet.	fracturing fluid gels such as guar are organic matters favoring bacterial growth. These bacteria break down the gelling agent, and viscosity can be reduced. Adding a biocide to the fracturing fluid kills these bacteria.
cross-linker	0.006	borate salts	used to cross-link polymers to provide higher viscosities with the least amount of the polymer.
gel	0.05	guar gum or hydroxyethylcellulose	it provides a better carrying efficiency than water to transport the proppant to the fractures.
pH-adjusting agent	0.01	potassium/sodium carbonate	maintains the effectiveness of other components, such as cross-linkers
acid	0.11	hydrochloric acid or muriatic acid	used to clean residuals resulting from drilling mud, cementing, or perforations.
scale inhibitor	0.04	ethylene glycol	prevents scale precipitation such as calcium carbonate.
corrosion inhibitor	0.001	*N*,*N*-dimethyl formamide	inhibits steel tubing corrosion of the tools, well casings, and tanks. Mainly used if acids are added to the fracturing fluid.
other	0.44		improve the performance of a fracturing fluid based on the properties of the formation

Analyzing the hydrocarbon production rates in approximately 293 wells in the Barnett Shale showed that a slick-water fracturing fluid (using a drag reducer) generally outperformed cross-linked and foam-fracturing fluids.^26^

#### 2.2.2 Polymers as a Transport Medium for Proppants in Fracturing Fluids

During hydraulic fracturing, a fracturing fluid (constituting a proppant of approximately 4.5% of the total weight of the fluid) is pumped at high pressures, causing the formation to crack open and form a network of fractures. The proppant propagates into the fractures by implosion and keeps the fractures open after the pressure is relieved. The hydraulic fracturing proppant is usually sand or a support agent such as coated ceramics. Proppant suspension by a viscosifier such as a polymer in the fracturing fluid is essential to transport the proppants into the created fractures. Hydraulic fracturing proppants are used based on their characteristics as follows:^27^

- Specific gravity is less than 2.0 g/cm^3^.
- Withstand closure stresses up to 20,000 psi.
- Chemically stable in brine at high temperatures up to 200 °C.
- Spherical.
- Have a narrow size distribution to get a uniform flow from the reservoir.

In slick-water fracturing fluids in shale reservoirs, the mechanism of proppant transport is rate-dependent. This is due to the fact that the polymer concentration is low in slick water, yielding a low viscosity, which makes it incapable of keeping the proppant suspended in the fracturing fluid. In this case, proppant transport is dominated by the proppant movement its self. On the other hand, most of the aqueous liquids used for fracturing fluids are gelled. Ideally, gelation of the fracturing fluids is achieved by a polymeric gelling agent. The gelled fracturing fluid maintains the proppants floating within the fluid and transport it to the fractures in the reservoir. In addition, a layer of high viscous polymer (filter cake) will form on the face of the fracture, which prevents further fracturing fluid leak-off into the formation rock.

**Figure 5:**
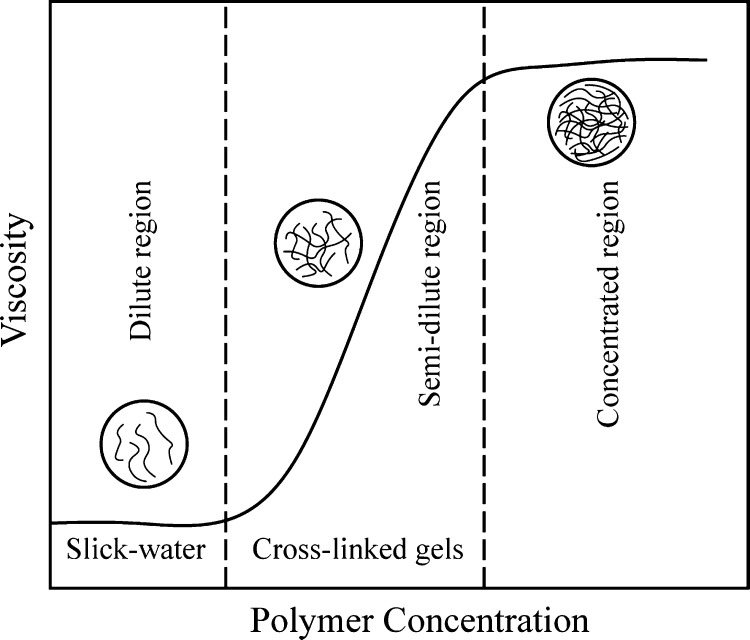
Schematic showing the effect of polymer concentration on viscosity.

The difference between gelled and slick-water fracturing fluids is the concentration of the polymers used in the fluid and the resulting viscosity. Figure 5 illustrates three typical concentrations regions used in gelled and slick-water fracturing fluids based on the yield viscosity. Below the dilute low-concentration region resides the slick water. Cross-linked polymers are typically located in the semidilute region. When the polymer concentration exceeds the concentrated region, the gel will be over-cross-linked, squeezing water out of the gel matrix. This phenomenon is called syneresis.^28^

Fluids comprising guar gums have been utilized since the late 1950s in fracturing fluids. The addition of guar thickens and viscosifies the fracturing aqueous fluid, which provides the fluid with the ability to transport the proppant to the fractures. Guar derivatives such as hydroxypropyl guar, carboxymethyl guar, hydroxypropyl cellulose, and carboxymethyl hydropropyl guar have also have been used due to their ability to cross-link, resulting in higher viscosities and optimum fracture geometry.^29^

Recent literature reported some polymers maintaining high viscosity and efficient carrying capacities. Li et al. (2014) used brine samples with high total dissolved solids and a high temperature of 270 °F. The cross-linked polymer solution maintained a 100 cP viscosity for more than 1 h and then started to decrease.^30^ The solution maintained a similar viscosity for more than 2 h when the temperature was at 250 °F. Interestingly, their study showed an 89% permeability of the targeted permeability of the proppant. This led to a good proppant pack arrangement caused by the better carrying capacity of the solution and improved settlement of the proppant. Monreal et al. (2014) also used a cross-linked polymer system to study the effectiveness of the carrying capacity of the fluid system.^31^ The fluid system was analyzed at a lower temperature of 150 °F with high salinity. The results gave a good solution stability for 1 h and a half with a viscosity of 150 cP. Another interesting study was reported by Gaillard et al. (2013) with an improved synthetic polymer structure with a surfactant. The synthetic polymer was prepared so that it has a resistive property to degradation by elevated total dissolved solids in brine. At an approximate temperature of 210 °F, the solution achieved a viscosity of 100 cP for more than 2 h. Gaillard et al. (2013) also reported that the viscosity was maintained at this value when the surfactant was added; however, the viscosity significantly dropped when the surfactant was not used.^32^

More advancements in technology and needs for cheaper and more reliable, high-temperature-tolerant, and less formation-damaging polymers revealed different types of cross-linked polymers. Polyacrylamide, polyacrylates, xanthan, and cellulose derivatives were implemented in fracturing fluids, showing better fracturing performance, especially for unconventional reservoirs. Boron, titanium, zirconium, and aluminum complexes are the cross-linking agents used to cross-link linear polymers and improve their transport efficiency and temperature tolerance.

Synthetic polymers (acrylamide-based polymers) are currently widely used in oilfield operations. Although polyacrylamide and hydrolyzed polyacrylamide are widely used in cross-linked fracturing fluids, there are some operating limitations at high temperature and salinity. Such harsh environments revealed the tendency to use other acrylamide base polymers such as 2-acrylamido-2-methylpropanesulfonic acid (AMPS).

### 2.3 Challenges of Polymer Application in Fracturing Fluids

Most polymers generally are susceptible to degradation to some degree by shear, conformational changes, and oxidation. The polymer degradation study is important to understand the drag reduction mechanism of the polymer. Therefore, the literature reported several studies on polymer mechanical, thermal, and high-salinity degradation.^33^ The reduction in fracture conductivity and adsorption of polymers in fracture and matrix are also a major concern in using a polymer as an additive in fracturing fluids. However, the latter phenomenon is less reported in the literature when modeling a fracturing fluid system. Thus, we will give a comprehensive review on the adsorption phenomenon.

#### 2.3.1 Mechanical Degradation

Mechanical degradation results from high mechanical stresses induced by high shear (flow velocities or elongation deformations) of the polymer. Mechanical degradation is an irreversible process leading to the breakage of polymer molecules.^34^

**Figure 6:**
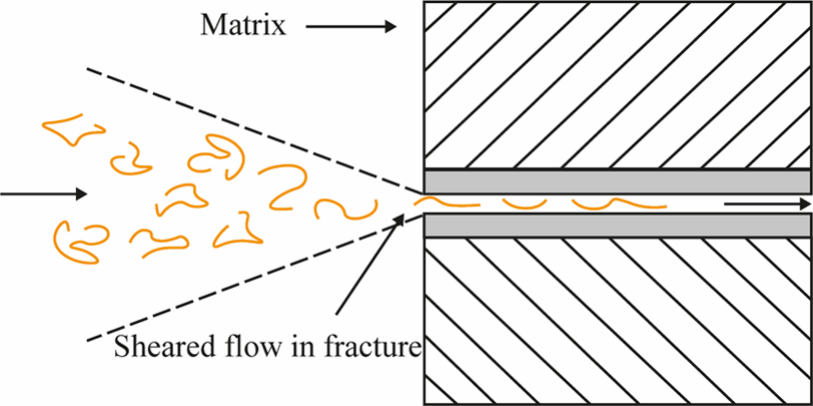
Mechanical degradation by the shear rate of polymers flowing in a fractured reservoir.

Figure 6 illustrates the mechanical degradation occurring in oil and gas applications due to high shear rates under turbulent flow through fractures.^35^

Mechanical degradation of polymer molecules at high shear rates uncoils the polymer, resulting in a significant loss of polymer viscosity and reducing its displacement efficiency and drag reduction capability. The use of polymers for fracturing is essentially bound with its rheology;^36^ for instance, polymer injection is limited by shear thickening behavior through associative pressure build-up that can result in wellbore fracturing or mechanical degradation of the polymer. Both polymer mechanical degradation and fracturing make the preassessment of polymer injectivity challengeable.

A synthetic polymer seems to be more sensitive to mechanical degradation than biopolymers. Comparing mechanical degradation for HPAM and xanthan viscosities with the shear rate, we found that xanthan showed an extreme shear stability because of its rigid rod structures, whereas HPAM with its flexible coil molecules appeared to be very sensitive to shear. More studies were dedicated to the comparison of mechanical degradation behavior for polyethylene oxide, polyacrylamide, xanthan, and guar gum. Initially, the synthetic polymers showed greater drag reduction performance efficiency, but as a result of shear, drag reduction of the synthetic polymers was reduced faster than the xanthan and guar. Overall, xanthan and guar are better shear-resistant polymers at high flow rates, but the drag reduction of xanthan and guar is smaller than that of synthetic polymers.^37^ Mixtures of synthetic polymers with xanthan/guar were also reported to have the possibility of improving mechanical degradation of the synthetic polymers. Habibpour (2017) showed that mixing HPAM with xanthan slightly improved the drag reduction stability of HPAM solutions.^38^

It is noteworthy to mention that polymer mechanical degradation becomes relatively severe at higher flow rates and lower permeabilities. This condition implies to the slick-water fracturing applications at high flow rates in low- and ultralow-permeability reservoirs. In shale reservoirs, for instance, the stress applied on the polymer is high, resulting in the breakage of the polymer chains and a severe reduction in the fluid viscosity. However, polymers with higher molecular weights can experience a molecular weight distribution after breaking the polymer chain, resulting in fragments with lower molecular weights. Seright (1983) reported the initial and final molecular weight of HPAM after alteration. The HPAM molecular weight distribution showed almost a 50% decrease of the HPAM molecular weight from 12 million Da to almost 7 million Da.^39^ Sorbie (1991) concluded that there is a critical specific molecular weight (Mc) for a given shear stress where no mechanical degradation will occur below the Mc.^40^ This shows that it might be more applicable to investigate the Mc and the molecular weight distribution rather than looking at the average molecular weight of a polymer when screening potential polymers for fracturing applications.

#### 2.3.2 Thermal Degradation

**Figure 7:**
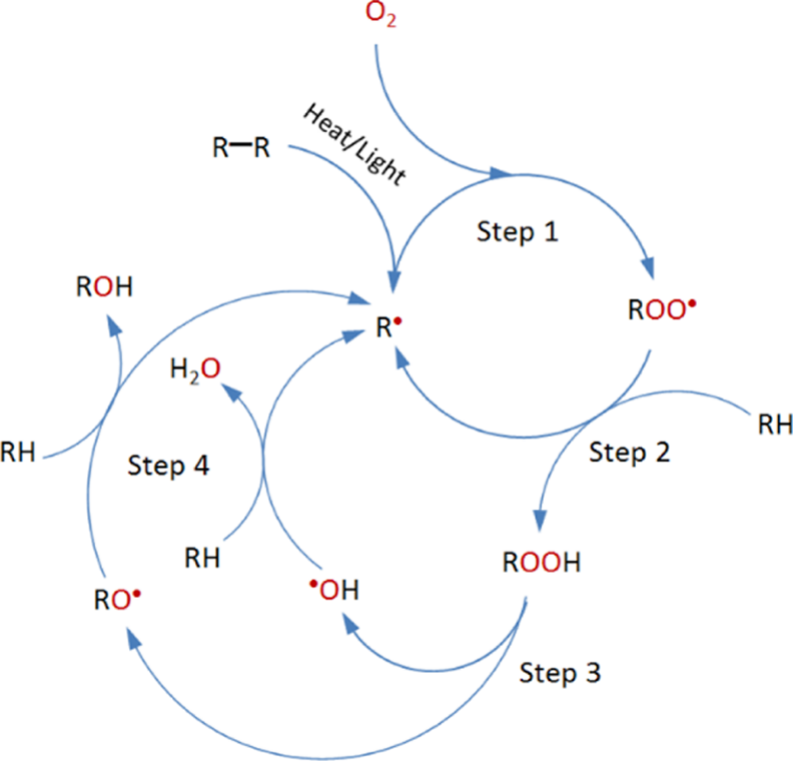
Polymer thermal degradation mechanism.

Thermal degradation is the deterioration of the polymer molecules caused by overheating. Increasing the temperature induces the components of the backbone of the polymer to break and react with one another, which changes the properties of the polymer. Figure 7 (from Polymer Properties Database) shows a general mechanism involving the three major steps of initiation, propagation, and termination for thermal degradation.^41^ The reaction generally starts at the chain ends (initiation), where the free radical is formed. Then, in the propagation stage, the monomers are sequentially detached from the main chain. Finally, in the last stage (termination), the polymer chain is completely depolymerized.

Polymers have been widely used in the oil and gas industry. Continuous significant attempts focused on the improvement of thermal properties of the polymer used for enhanced oil recovery and fracturing of deep and high-temperature unconventional reservoirs. Guar gum is the most known polymer in fracturing fluids. However, it is not stable at high temperatures of (>350 °F). Due to the need to fracture reservoirs with higher temperatures, researchers are investigating various synthetic polymers. Holtsclaw and Funkhouser (2010) reported the use of a terpolymer of AMPS and acrylamide showing a viscosity of 700 cP and a higher temperature tolerance (at temperature higher than 450 °F). Table 3 shows a summary of some of the reported polymers used in fracturing fluids with their maximum operating temperatures in the ascending order.

**Table 3:** Polymers Used in Fracturing Fluids with Their Maximum Operating Temperatures

polymer	maximum temperature (°F)	refs
linear biopolymer	200	(42)
hydroxypropyl guar	225	(43)
synthetic PAM	450	(44)
AMPS	>450	(45)

#### 2.3.3 High Salinity

The complex nature of shale reservoirs and a formation water salinity higher than 100 000 ppm makes it a challenge to find a suitable polymer for the fracturing fluid. Most water-soluble polymers such as polyacrylamide tend to hydrolyze/degrade in high salinity (ions such as Na^+^, K^+^, Ca^2+^, and Mg^2+^). At critical concentrations of saline, the polymer might flocculate and precipitate. Therefore, a polymer with good salt resistance is desirable when fracturing in such high-salinity shale reservoirs.

Moreover, in the recent literature, interesting results were reported from hydrophobically modified polymers (associative polymers), which can offer a great tolerance at higher salinities. Associative polymers are structured in a way that the backbone of the polymer is attached to both hydrophobic and hydrophilic moieties.^46^ Molecules/intermolecular associations form an amphiphilic structure, thereby enhancing the fluid performance in high-salinity reservoirs.

**Figure 8:**
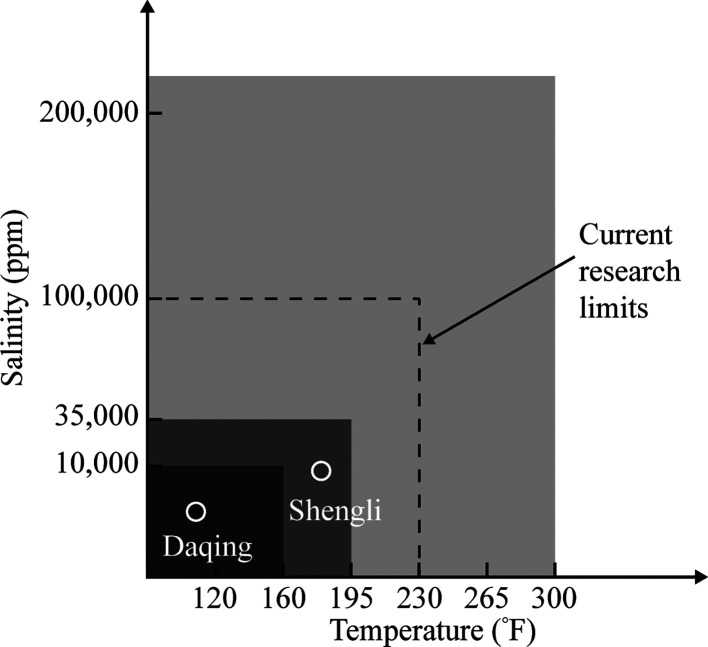
Temperature and salinity window for chemical stability.

Both temperature and salinity define the applicability of the chemicals in general and the polymer in particular used in fracturing fluids. Figure 8 shows the window for which polymer stability becomes more challenging at a given salinity and temperature.^47^ At high temperatures and high salinities, guar and xanthan showed a significant degradation. Sulfonated polyacrylamide polymers are reported to yield better stability at high temperatures and salinities.^48^ For instance, Vermolen et al. (2011) used a polyacrylamide base functionalized with the AMPS and *n*-vinyl pyrrolidone (*n*-VP) monomers at high temperature and salinity conditions. The AMPS monomer was used to promote the thermal stability of the mixture, and *n*-VP monomers were used to protect the polymer from hydrolysis at higher temperatures and promote tolerance to divalent ions.^49^ Thus, developing new synthetic polymers that are stable at high temperatures and high salinities will improve proppant transport and settling at the fractured faces.

#### 2.3.4 Reduction of Fracture Conductivity

As discussed in the previous section, a fracturing fluid needs to have the ability to carry the propping agent. The addition of polymers and cross-linking them with a metal ion increases the fluid viscosity. The type and concentration of the polymer and cross-linking agent and temperature will determine the viscosity of the fracturing fluid. After successful placement of the proppant, the gelled fluid needs to flow back to the wellbore to obtain the highest possible fracture conductivity, enhancing the production rate from a well. Cross-linked polymers are designed to break down through degradation by the addition of chemical breakers. However, some insoluble residue from the broken gels is left in the fracture, causing a significant reduction in the fracture conductivity. Hence, guar is composed of mannose and galactose (soluble in water) in the ratio of 1.6:1 and 1.8:1 respectively, with an eccentric distribution.^50^ A helix of polymannose (insoluble in water) can form from as few as six adjacent unbranched mannose units,^43^ causing 6–10% (w/w) insoluble residue.^50^ In addition, giving a long time to the gel breaker might cause more residue due to the formation of helices resulting from the incongruous breaking of the polymer.^43^ This can be one of the main reasons for investigating new synthetic polymers with better solubility in water to be utilized for fracturing fluids.

The use of cross-linked polymers generally results in the deposition of fibrous materials within the grains of the proppant pack, which is then glued together, resulting in a significant reduction of fracture conductivity.

Reduction of the fracture conductivity by the polymer residue results in a reduction of the effective fracture length. The effective fracture length is the part of the fracture which is cleaned up and contributes to the production rates. The ability to clean up the fracture depends on the conductivity ratio (*C*_r_), dimensionless fracture conductivity, of the fracture, as shown in eq 1(43)
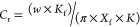
1where w is the fracture width (ft), *K*_f_ is the fracture permeability (mD), *X*_f_ is the fracture length (ft), and *K* is the formation permeability (mD). A conductivity ratio value of approximately 8 or higher suggests that the fracturing fluids need to be cleaned up from the proppant pack. Similarly, a conductivity ratio value of 10 or greater requires filtrate (invaded zone filtrate at fracture faces) cleanup. The conductivity ratio or dimensionless fracture conductivity is an important design parameter for a fracture treatment job in the industry.

It is noteworthy to mention that the effective fracture length is significantly affected by the properties of the fracturing fluid. This means that in situ fracture conductivity is reduced by the polymer gel left in the proppant pack, which is still a concern in the industry. Therefore, it is important to design the right fracturing fluid rheology, which determines the width of the fracture and net fracture pressure, increasing the process of propped fracture cleanup. Hence, higher viscosities are governed by the addition of polymers and cross-linked gels with proper breakers.

Although cross-linked polymers result in higher formation of the post-cross-link breakage residue, they result in a better fracturing geometry and less water volume loss. Low polymer concentrations yield lower fluid viscosity and poorer proppant transport and narrower widths of fractures. The low-viscosity fracturing fluid will penetrate deeper in the formation (micro- and nanofractures), the proppant will settle because of fluidization and deposition, and a monolayer of the proppant at the fracture face forms, causing a more complex fracture geometry.

#### 2.3.5 Gel Breakers

Breakers are chemicals that are able to break the high-molecular-weight cross-linked polymers into smaller molecules. Breakers are used to degrade the polymer gel to clean up the fracture and provide a better fracture conductivity. Chemical breakage of cross-linked polymers is difficult because of three reasons:^51^

**Figure 9:**
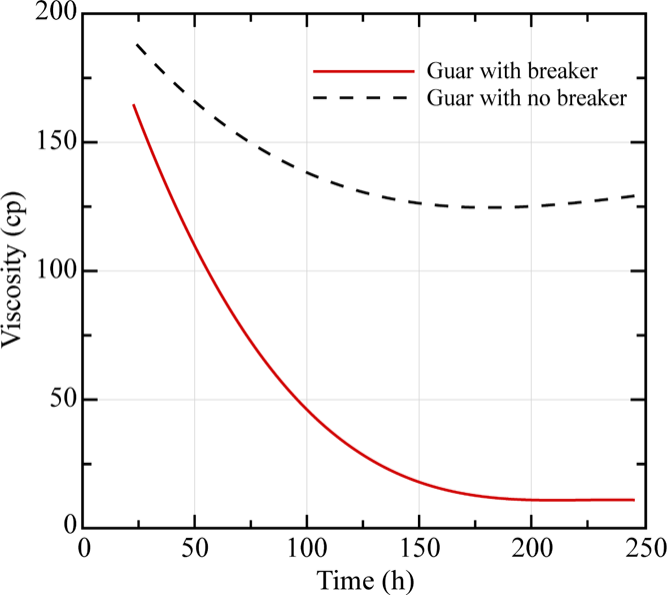
Effect of a breaker on guar viscosity with time.

- Breakers need to attack the backbone of the polymer.
- Breakers need to react with the cross-linked bond to the polymer. However, the fluid is pumped with a strong buffer, providing the most stable pH condition for the cross-linked polymer, which makes reacting to the breakers more difficult.
- A slow breakdown reaction is needed to obtain fracturing fluid stability for adequate proppant placement, as illustrated in Figure 9.^52^

Significant damage in the proppant permeability may also result from the inappropriate/ineffective use of the breakers.

The breaker is either premixed with the fracturing fluid before injecting the fluid downhole or sent after fracturing. There are mainly two types of commonly used breakers for cross-linked polymer systems: oxidizers and enzymes.^13^

Oxidizers are widely used in the fracturing fluid. Oxidizers such as persulfates and peroxides are reactive species that typically decompose and produce free radicals. The free radicals will attack the polymer chains and degrade them into smaller polymer molecules. A low temperature yields a slow free radical generation rate, resulting in slower breaking times. However, the oxidizers can be coupled with certain initiators or catalysts to accelerate the breaking process.

It is noteworthy to mention that the fracturing fluid may also contain dissolved oxygen which can also act as a breaker. This type of breaker is not suitable to completely degrade the polymer gel for cleanup purposes, but it can compromise the fracturing fluid viscosity during the pumping time. Therefore, high-temperature fracturing fluids are accompanied with stabilizers such as sodium thiosulfate to reduce such effects.

Enzymes are also used for breaking polymer gels in fracturing fluids. Enzymes are catalysts developed by organisms to achieve specific functions related to cellular metabolism processes. Various enzymes produced by certain fungi and bacteria are able to attack polymers such as guar. Unlike oxidizers, enzymes reduce the polymer molecular weight without being consumed when attacking the polymer gels. Moreover, enzyme breakers are typically used in acidic media at low temperatures below 60 °C.^53^

It can be inferred from the previous discussion that enzyme breakers are preferred in the fracturing fluids because they are miscible, environmentally benign, and easy to handle compared to oxidizers. Unlike oxidizers, enzymes are catalysts, so they are not consumed and cause an insignificant damage to the fracturing equipment. Moreover, enzymes are reported to have a better breaking efficiency of guar polymers compared to oxidizers, which leave more residues. However, enzymes are sensitive to high pH and temperature, which makes oxidizer breakers an alternative option in fracturing.

An important concept about breakers is that breaking polymer links between mannose groups directly reduces the average molecular weight, which reduces the solution viscosity. On the other hand, using breakers to break the polymer links of individual galactose–mannose will not result in a significant change in the viscosity. Nevertheless, more than six consecutive chains of galactose can be removed from this breakage, resulting in helix formation and, hence, precipitation, as highlighted in the previous section. Therefore, gel breakers have to simultaneously break the polymer backbone and side chains to maintain a balanced ratio between galactose and mannose. Moreover, the breaker efficiency is improved with the development of encapsulated breakers, allowing the use of high breaker concentrations. These encapsulations are crushable material films acting as barriers between the fracturing fluid and the breaker. However, at higher temperatures, stronger coatings need to be developed.

#### 2.3.6 Polymer Adsorption

As indicated in the previous sections, polymers are largely used in fracturing fluids as fraction reducers or proppant carriers due to their ability to increase fluid viscosity. These polymers will normally interact with the shale surfaces. Typically, the interaction between the long-chain polymer with the rock surface due to cation exchange or change in entropy is referred to as adsorption.^54^ Polymer adsorption leads to the binding of the polymer molecule onto the rock surface mostly due to physical adsorption (hydrogen bonding and van der Waals forces). In addition, polymer adsorption might take place on the surface of the injected proppant.

**Figure 10:**
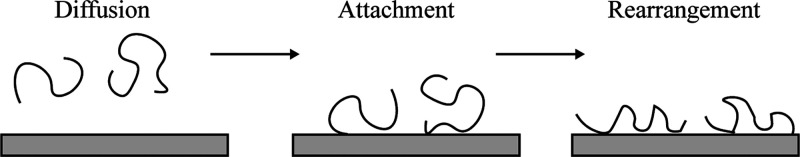
Steps for polymer adsorption on a solid surface.

Polymer adsorption is commonly calculated by polymer concentration depletion in a solution making contact with a solid surface.^55^ There are generally three steps for polymer adsorption: diffusion, attachment, and rearrangement of the polymer molecules, as illustrated in Figure 10.^56^ First, diffusion of the polymer molecule occurs, after which the polymer is transported from the bulk solution to the rock surface. Then, the polymer is attached to the rock surface (hydrogen bonding and van der Waals forces). Finally, relaxation of the polymer molecules (rearrangement) occurs at the rock surface adsorption sites.

The adsorption of the polymer to the solid surface with respect to time (adsorption rate) can be calculated usingeq 2^56^
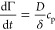
2where *D* is the polymer diffusion coefficient (m^2^/s),  is the stagnant layer thickness just above the surface (μm), and *c*_p_ is the polymer concentration (ppm). Typically, to reach saturation at the solid surface, adsorption takes no more than about 5 min (adsorption rate ≈ 0.005 mg/m^2^s). However, adsorption takes a longer time to reach the equilibrium. This can be explained by the time taken for polymer molecule rearrangements at the solid surface. Thus, approximately 24 h are recommended for polymer adsorption measurements to reach the equilibrium.

Polymer adsorption depends on various parameters such as polymer concentration and molecular weight, salinity, ionic strength, solid-to-liquid ratio, and temperature.^54,57^ Moreover, higher polymer adsorption occurs if the interaction between the polymer and solvent is weak. It is significant to predict polymer adsorption in shale as polymer adsorption is generally irreversible.^58^ This will lead to the reduction of fracture conductivity and proppant carrying capacity. Xiong et al. (2018) conducted an adsorption study of the PAM polymer on a Marcellus Shale sample and reported a significant change in the peak area in the size-exclusion chromatograph before and after adsorption.^59^ This change in the peak showed a decrease in the PAM concentration from 717 to almost 105 ppm, which is approximately 85% loss of the original PAM concentration. Xiong et al. indicated that this loss was due to adsorption on shale and chemical degradation.^59^ Their results show how it is important to estimate polymer adsorption before field applications. Partial retention of the polymer-based fracturing fluid increases the formation damage and reduces the fracture conductivity after the fracture closes. As discussed, polymer adsorption causes this retention mainly. Reduction of polymer adsorption around the fracture faces in the formation is also an area of further research, which is in progress. Nanofluid-based polymers in fracturing fluids are also currently being researched, which will reduce the polymer adsorption, formation damage, and increase the oil mobility.^60^

##### 2.3.6.1 Polymer Adsorption in Slick-Water Fracturing Fluid Systems

**Figure 11:**
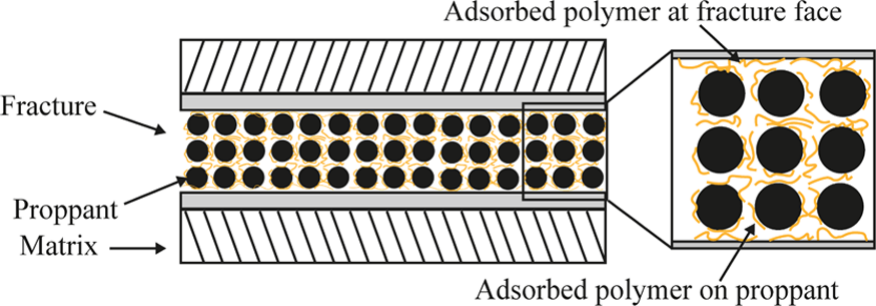
Schematic showing polymer adsorption on the proppant and fracture face.

Hydraulic fracturing and horizontal good technology made development and production from the extremely tight permeability shale reservoirs possible. The slick-water system, in particular, is widely used as a fracturing fluid in almost 80% of the shale fracturing projects worldwide.^61^ Slick water containing a small concentration of drag reducers (polymer) is used to reduce the fracturing fluid friction with the tubing, as explained in previous sections. Even though polymers are used in low concentrations, they form a chemical bond with the shale minerals, specifically the negatively charged clays. This leads to the adsorption of the polymer at the wall of the fracture face, formation, and proppant matrix, as shown in Figure 11.^62^ Hence, the adsorbed polymer may cause a significant reduction of the fracture microcracks (fractures) and matrix permeability because the pore radius of shale reservoirs and the hydrodynamic size of the polymer molecules are both in nanoscale.

##### 2.3.6.2 Polymer Adsorption in Gelled Fracturing Fluid Systems

There are two concerns over using a cross-linked gel in fracturing fluids: cross-linked gel residue and polymer adsorption. The polymer gel residue may reduce fracture conductivity, as indicated in previous sections. Significant reduction results in permeability because of the cross-linking of the polymer solution. Interestingly, reduction in permeability does not show a direct proportionality with increasing polymer concentration. This means that this reduction is a result of a combination of polymer reduction and the polymer residue. Marpaung et al. (2008) used dynamic fracture conductivity experiments to measure the polymer gel residue residing in the fracture.^63^ Their results showed that the fracture conductivity measured with the standard loading of the proppant using static procedure was twice as high as that of the dynamic test. This was explained by the higher creation of the polymer gel filter cake in the dynamic test.

On the other hand, polymer adsorption may also occur at the wall of the fracture face, formation, and proppant matrix, as explained in the previous section. This, in fact, has a great impact on the performance of the cross-linked polymer efficiency. The formation and performance of the polymer gel are a function of the concentration of the polymer. Because of polymer adsorption, the concentration of the polymer can be significantly reduced, which will reduce the polymer gel carrying efficiency. Thus, the gel strength can be weakened and the time required for gelation can be extended because of polymer adsorption. This kind of behavior was reported in the literature as a possible reason for weaker gel formation.^64^Table 4 shows some of the significant reported polymer adsorptions on shale clays.

**Table 4:** Polymer Adsorption in Clay Minerals

polymer concentration (ppm)	adsorbate	adsorbent	polymer adsorption (μg/g)	refs
10–330	guar gum	talc	850–2100	(65)
200–600	poly(vinyl pyrrolidone)	montmorillonite	500–3000	(56)
	polyacrylamide	K-smectite	300–1000	(66)
0–1200	anionic PAM 836A	illite	0–14 000	(67)
		smectite	0–10 000	
		kaolinite	0–8000	
0–1200	nonionic PAM 903N	illite	0–35 000	
		smectite	0–70 000	
		kaolinite	0–14 000	
0–1200	cationic PAM 494C	illite	0–10 000	
		smectite	0–18 000	
		kaolinite	0–11 000	
100	cationic guar	illite	≈83 000	(68)
		montmorillonite	≈82 000	
100	nonionic guar	illite	≈80 000	
		montmorillonite	≈78 000	
100	anionic guar	illite	≈77 000	
		montmorillonite	≈58 000	
100	cationic PAM	illite	≈79 000	
		montmorillonite	≈50 000	
100	nonionic PAM	illite	≈50 000	
		montmorillonite	≈10 000	
100	anionic PAM	illite	≈60 000	
		montmorillonite	≈5000	

The success of fracturing application depends on providing an efficient suspension of the proppant in the fracturing fluid. This will ensure proppant delivery and settlement in the induced fractures. Therefore, designing a fracturing fluid viscosity for the proppant suspension is crucial. Polymer adsorption can result in polymer concentration reduction by as much as 85%,^59^ which results in extreme reduction of the solution viscosity. This will significantly affect the transport and settlement of the proppant in the fracturing fluid, resulting in the reduction of fracture permeability or a total closure of these fractures. Thus, polymer adsorption is an essential parameter that determines the feasibility of a fracturing application.

It is noteworthy to mention that the mechanisms of proppant delivery and settlement for slick-water fracturing are different. A very small polymer concentration (5–10 pptg) is used in slick-water fracturing to reduce friction with the tubing, as indicated in previous sections. This concentration is not enough to increase the viscosity to such a value that is able to suspend the proppant. In this case, the settlement of the proppant is faster and the transport of the proppant is rate-dependent, caused by the movement of the fracturing fluid itself. However, polymer adsorption still exists in this case, resulting in a significant reduction of the permeability of the fractures.

## 3 Conclusions

This paper presents a perspective review of polymers as drag reducers and transport media in water-based fracturing fluids. This paper aims to provide a comprehensive reference for researchers investigating the polymers in fracturing fluids and field practitioners designing a fracturing fluid for their project. The study also provides a review of the applications, developments, and limitations of polymers in fracturing fluids. The following can be concluded from this study:

- A shale formation has a significant role in oil and gas, serving as hydrocarbon source rocks, seals, and reservoirs in shale oil/gas plays.
- Natural gas production has considerably increased with the advancement in hydraulic fracturing and horizontal drilling techniques in the past 2 decades.
- It is important to design a suitable fracturing fluid with a polymer that is adequate for particular shear, temperature, and salinity conditions. This is because polymers are susceptible to degradation by shear, conformational changes, and oxidation.
- In slick-water fracturing fluids, polymers with favorable higher molecular weights are added in a small quantity at a concentration of 5–10 pptg to the fracturing fluid to reduce the friction of the fracturing fluid inside the tubing.
- Linear polymers can form a single macromolecule with a network structure via cross-linking using a cross-linking agent, yielding a high viscous fluid that is able to efficiently transport the proppant to the fractures in the reservoir. Wider fractures can be obtained from the cross-linked gelled polymer compared to the slick-water fracturing fluid system due to the higher viscosity.
- Water-based slick-water and gel fracturing methods have proven to be pioneer methods among the various fracturing methods.
- Synthetic and associative polymers are currently widely used in oilfield operations, offering a great tolerance at higher shear, temperature, and salinities.
- Although polymer adsorption is not well considered in designing a fracturing fluid, it is a significant parameter that might impact the performance of the polymer efficiency to reduce the friction and transport of the proppant.

## 4 Future Outlook of the Use of Polymers in Fracturing Fluids

Polymer losses in the formation after hydraulic fracturing are a major concern in well stimulation operations. The retained polymer in the fracture as discussed in the previous sections results in a considerable reduction in proppant pack permeability, which reduces the effectiveness of the fracture treatment. Willberg et al. (1997) reported that as little as 29–41% of the polymer used in fracturing fluid was collected during the flow-back period from the well.^69^ Therefore, researchers attempt to find an alternative for water-soluble polymers.

Successful applications of the VES in enhanced oil recovery and frac-pack applications resulted in the development of surfactant-based fluids for hydraulic fracturing. The advantage of the VES fluid system is its simplicity in the operational preparations of the fluid and types of equipment at the well site. However, VES-based fracturing fluids have limited conditions in the hydraulic fracturing of oil and gas reservoirs with temperatures below 240 °F.^70^

The VES in fracturing fluids uses the fundamental characteristics of a surfactant, that is, the presence a hydrophobic and a hydrophilic group in a molecule and their molecular size, which is almost 5000 times smaller than the molecules of a guar. This feature allows the surfactant molecule to have an opposing tendency for solubility, hydrophilic (water-soluble ionic group), and hydrophobic (oil-soluble hydrocarbon chain). When the surfactant is dissolved in an aqueous solution, a micellar structure is formed using a small group of surfactants. These micelles have a geometry similar to the polymer molecules, which provides the solution with distortion resistance, allowing the increase in viscosity and endowing viscoelastic properties to the fluid.

Chen et al. (2005) reported a novel VES fluid compatible with supercritical carbon dioxide (CO_2_).^71^ The polymer-free gelling agent developed contained a mixture of surfactants that increases the viscosity of the aqueous phase and provided stability for the liquid–liquid interface of the supercritical CO_2_ and the aqueous phase. The proposed fluid offers the advantage of VESs which are solid-free, with CO_2_ providing enhancement for cleanup in depleted reservoirs. However, the fluid system is stable at temperatures up to 230 °F and only operated at a low salinity of 2% KCl.

Recent studies aimed to improve the rheological characteristics of the foam and increase the capacity of the proppant settling during hydraulic fracture. Yan et al. (2016) developed a novel reusable VES fracturing fluid^72^ which was environmentally friendly using a CO_2_-responsive surfactant. The study showed that controlling the gelling or breaking the gels can be achieved by simply regulating the surfactant pH. Khair et al. (2011) reported anionic surfactant fracturing fluid properties and field applications. The viscosity of the fluid was determined using a rotational viscometer, and the liquid elasticity was determined using a rheometer.^73^ The fracturing fluid was thermally stable at temperatures above 195 °F with minimal damage to the formation with 97% estimated returned permeability. Gemini VESs were also investigated that offered high viscosity stability in the presence of KCl at an elevated temperature of 320 °F.^74^ The results suggested that KCl assists the aggregation and entanglements of the micelles, rendering tighter packing of the surfactant micelles. Nevertheless, the gemini VES molecular structure stability determines this enhancement process of the micelles. A gemini cationic VES-based fracturing fluid can maintain a higher viscosity over 50 cP and at a higher shear rate of 100 s^–1^ and a temperature of 250 °F and is also found to be useful for wellbore clean-outs.^75^

VES fracturing fluids are used because of their principle advantage, whereby they are easy to prepare at well sites, minimal formation damage, and hence high proppant pack conductivity. Additionally, no breakers, no cross-linkers, nor other chemical additives are required, which are essential in polymer-based fracturing fluids. However, besides the operating temperature limitation, surfactant-based fracturing fluids have a very poor property of proppant transport and low leak-off control, and at high pumping rates, the friction is significantly severe. Although commercially available additives can improve friction, the formation may alter the fluid composition and proper stimulation cannot be achieved. It can be concluded that polymers offer a better carrying ability and stability under harsher operation conditions than the current polymer-free alternatives. A better insight would be a trend to find a method to enhance the polymer residue cleanup and mitigate polymer adsorption equivalent to all the efforts to improve polymer performance for enhanced oil recovery.
